# Combining two open source tools for neural computation (BioPatRec and Netlab) improves movement classification for prosthetic control

**DOI:** 10.1186/s13104-016-2232-y

**Published:** 2016-08-31

**Authors:** Cosima Prahm, Korbinian Eckstein, Max Ortiz-Catalan, Georg Dorffner, Eugenijus Kaniusas, Oskar C. Aszmann

**Affiliations:** 1 Institute of Electrodynamics, Microwave and Circuit Engineering, Vienna University of Technology, Gusshausstr. 25, 1040 Vienna, Austria; 2 Department of Radiology and Nuclear Medicine, Medical University of Vienna, Lazarettgasse 14, 1090 Vienna, Austria; 3 Division for Biomedical Engineering, Department of Signals and Systems, Chalmers University of Technology, Campus Johanneberg, 41296 Gothenburg, Sweden; 4 Center for Medical Statistics, Informatics and Intelligent Systems, Medical University of Vienna, Spitalgasse 23, 1090 Vienna, Austria; 5 CD Laboratory for Extremity Reconstruction, Division of Plastic and Reconstructive Surgery, Medical University of Vienna, Währinger Gürtel 18-20, 1090 Vienna, Austria

**Keywords:** Prosthetics, Upper limb amputation, Machine learning, Pattern recognition, Neural computation

## Abstract

**Background:**

Controlling a myoelectric prosthesis for upper limbs is increasingly challenging for the user as more electrodes and joints become available. Motion classification based on pattern recognition with a multi-electrode array allows multiple joints to be controlled simultaneously. Previous pattern recognition studies are difficult to compare, because individual research groups use their own data sets. To resolve this shortcoming and to facilitate comparisons, open access data sets were analysed using components of BioPatRec and Netlab pattern recognition models.

**Methods:**

Performances of the artificial neural networks, linear models, and training program components were compared. Evaluation took place within the BioPatRec environment, a Matlab-based open source platform that provides feature extraction, processing and motion classification algorithms for prosthetic control. The algorithms were applied to myoelectric signals for individual and simultaneous classification of movements, with the aim of finding the best performing algorithm and network model. Evaluation criteria included classification accuracy and training time.

**Results:**

Results in both the linear and the artificial neural network models demonstrated that Netlab’s implementation using scaled conjugate training algorithm reached significantly higher accuracies than BioPatRec.

**Conclusions:**

It is concluded that the best movement classification performance would be achieved through integrating Netlab training algorithms in the BioPatRec environment so that future prosthesis training can be shortened and control made more reliable. Netlab was therefore included into the newest release of BioPatRec (v4.0).

## Background

Performance of machine learning algorithms are constantly compared with one another to improve the classification of motion based electromyographic (EMG) signals in order to control a prosthetic device. Since mechatronically the number of controllable joints has increased and simultaneous control is replacing sequential control, the limiting factor to be improved in the future is the human-machine-interface.

Since this improvement is a challenging task, conventional control strategies for myoelectric prostheses have not changed much over decades [1]. Two bipolar electrodes are placed on one of the amputee’s residual muscles each and a sufficiently high amplitude of the EMG signal triggers a threshold detection method which then activates a prosthetic movement [2, 3]. This conventional control strategy, even though proven to be reliable [3, 4], turns out to be tedious and slow when there is more than one joint to be controlled. To change a currently selected degree of freedom (DoF), e.g. from wrist to elbow, both electrodes need to be activated simultaneously. This manual switching between joints is far from the natural movement of a hand and prostheses users are quickly frustrated with the limitations of their device [3].

The machine learning approach for prosthetic control makes use of an array of electrodes instead of just two control sites and considers patterns of EMG activation to increase the number of motion classes. EMG pattern classification in its early stages focused mainly on controlling one DoF at a time [5], which was not very different from the user experience of the conventional control. In the last years, however, pattern recognition has been extended to concurrent classification of motion intent [6–8]. Each activation pattern received by the electrode array around the amputees residual muscles corresponds to either an individual prosthetic motion, or a simultaneous one that involves multiple DoF. This way, the pattern recognition approach enables simultaneous control and thus promotes natural interaction with the environment.

To achieve a high classification accuracy, several types of classifiers for myoelectric prosthetic control have been examined such as artificial neural networks (ANN) [9–11], linear discriminant analysis (LDA) [12–15], support vector machines [16], k-nearest neighbour clustering [17] and unsupervised clustering [18]. High classification accuracy is important to prevent misinterpretations of the prosthesis user’s motion intent.

Previous studies proposed a multi-layer perceptron (MLP) classification strategy that outperformed an LDA based approach and improved the state-of-the-art classification [19]. However, the error rate was still high with 5 % which has also been reproduced in this study. In another study, accuracies of 96.3 % have been reached with a Gaussian mixture model (GMM) based classifier compared to LDA with 95.6 % [13]. They also compared GMM to MLP but did not specify the MLP training algorithm and network properties. Since the MLP only achieved an accuracy of only 95.4 %, it suggests they did not use an optimized training algorithm such as scaled conjugate gradient (scg). Another research group achieved accuracies of up to 97.6 % comparing a self enhancing linear approach with a standard LDA that reached 94.1 % accuracy [15], which corresponds to the LDA accuracy achieved within this study. However, because both groups used their own data set which is not accessible, the results are not directly comparable.

This study aims to evaluate and compare the performance of four open-source, Matlab-based pattern classifiers taken from BioPatRec and Netlab on correctly categorizing offline EMG signals across different movements.

The classifiers used in previous studies by BioPatRec are (amongst others) LDA and MLP. Therefore this study extends the previous studies [7, 20] using optimized algorithms for MLP and generalized linear models (GLM) taken from the Netlab toolbox to improve classification accuracy. Regularly, many studies use their own data set to test algorithms, which limits generalization of their results across methods. To grant comparability across methods, this study works on the same openly available data set [21].

BioPatRec is a useful environment for pattern classification already operated in a clinical setting [22]. Because training the classifier took very long and the resulting accuracy looked improvable, other options to increase classification accuracy were considered. To keep the initial set-up, these other options should consequently be implemented into the BioPatRec environment. The goal of this study is to find the algorithm with the highest classification accuracy and least computational complexity and therefore lowest training time that increases the performance of a myoelectric prosthetic controller. Since the newest release of BioPatRec, Netlab has been successfully integrated.

## Methods

A linear and non-linear classifier each, taken from both toolboxes BioPatRec and Netlab were compared with one another regarding offline movement classification performance and training time. The fastest algorithm within BioPatRec was LDA, which also is often used by researchers. The classifier reaching the highest accuracy within BioPatRec was MLP. Both classifiers were compared with Netlab’s highly optimized training algorithms iteratively reweighted least squares (irls) and scaled conjugate gradient for GLM and MLP.

The main benefit of linear methods such as LDA and GLM is their low complexity and quick training [5, 12–14, 23]. Artificial neural networks such as MLP depend on specific training algorithms and are inherently capable of simultaneous predictions. They can still be cost effective despite their increased complexity [10, 11, 19, 24].

To enable repeatability, the algorithms were compared using an open access data set from the BioPatRec data repository. This set has already been used for comparing different classifiers in previous evaluations [25, 26]. Three DoF are available for motion classification and used for individual and simultaneous control strategies, resulting in 26 possible movement labels and one no-movement label, in which no intentional EMG signal occurs. The resulting algorithm accuracy reflects the correspondence of the instructed user motion intend to the recognized movement by the classifier. The higher the accuracy the more reliable is the movement recognition. It is calculated by the number of correct classifications divided by the overall number of performed classifications.

Netlab is an open source software toolbox with highly optimized training algorithms for data analysis, neural computation and neural network simulations which requires the Matlab environment to run [27–29]. The library includes a variety of implementations for data analyses and neural network simulations.

BioPatRec is an open source Matlab-based research environment for development and evaluation of pattern recognition algorithms for prosthetic control. Matlab’s statistics toolbox is required. It provides tools for data acquisition, signal processing, feature selection and extraction, pattern recognition and real-time control and was developed by one of the authors—Max Ortiz-Catalan [20].

### A. Data source and treatment 

The data set which was used for comparing classification consists of 17 EMG recordings, one from each of the 17 healthy participants (mean age of 25.9 ± 4.9 years) and is available within the BioPatRec data repository [21]. Data was recorded by eight bipolar surface electrodes attached around the forearm. From each of the eight electrode channels, four time domain signal features (mean absolute value, waveform length, zero crossing and slope sign change) were extracted from 121 fixed time windows for each movement (200 ms with a time increment of 50 ms) which are used as feature vectors to feed the classifiers [20]. During recording the participant received a visual indication to perform a specific movement. Each movement was repeated 3 times with a recording time of 3 s. The first and last 15 % of that recording were removed to make sure only isotonic contraction will remain. Thus, a total of 121 windows were generated per movement. Out of these 121 windows per movement, 72 were randomly selected as training set and 49 windows were set aside as a validation set to evaluate generalization after the training phase and were not previously used for training. Performance accuracy was averaged over 100 iterations per subject (cross-validation), with a different random splitting into training and test set. All data was pre-processed and treated within BioPatRec.

Without normalization, features with different standard deviations would be weighted differently, error surfaces would be distorted and learning algorithms would not sufficiently converge. As a preliminary measure and because BioPatRec offers the possibility to choose among different normalizations, two commonly used linear normalizations in data processing were compared across all models: standard normalization $\mu$ = 0, $\sigma$  = 1 and a transformation to [−1;1] called “midrange 0 range 2”. The comparison table can be found in the supplementary material. Due to equal or better performance of the standard normalization all data was treated with standard normalization.

Netlab is able to use multiple processor cores with an average workload of 80–100 % on a quadcore CPU. BioPatRec MLP uses one processor core with an average workload of 25 % on a quadcore CPU. The PC used in this study has an i5-2500K processor running at 3.3 GHz when multiple cores are occupied, and at 3.7 GHz when just one core is active.

### B. Movement classification 

There are 27 possible labels for classification which derive from six individual hand and wrist movements as well as their simultaneous combination and an additional no-movement state the participants were instructed to perform. The individual movements were hand open/close, hand flexion/extension and wrist pronation/supination. Possible output types are (a) one single active unit, that can either represent one individual movement or a simultaneous movement consisting of a combination of individual movements; and (b) multiple active units in which each output unit represents one individual movement. Simultaneous movements are a combination of multiple output units.

### C. Network architecture of BioPatRec and Netlab

BioPatRec was used as a platform to execute both its own inherent classifiers (LDA and MLP) and the additionally implemented Netlab classifiers (GLM and MLP). Both models employ the two forms of output types mentioned above (single and multiple), that are implemented in Netlab with softmax and logistic output functions respectively. The advantage of using a softmax output function is an increase in accuracy, when there is a single classification problem.

*Linear classification method of BioPatRec and Netlab * BioPatRec’s LDA is taken from the Matlab statistics toolbox. Because this LDA can not generate multiple outputs as a single classifier, a BioPatRec inherent multi class problem approach was used that consists of one LDA for each degree of freedom. The individual LDAs possess four output neurons, two are for classifying the agonistic and antagonistic movement of the respective DoF, one is for every other movement and the last one is for a no-movement state.

The Netlab GLM uses scaled conjugate gradient or iteratively reweighted least squares as training algorithm. Regarding multiple outputs, Netlab GLM also uses the same configuration as BioPatRec’s LDA with one net for each degree of freedom and four output units.

*Non-linear classification method of BioPatRec and Netlab* The BioPatRec MLP is a feedforward artificial neural network (ANN) with backpropagation as supervised learning and gradient descent as training algorithm with a logistic activation function [20]. The standard configuration of BioPatRec consists of 32 input neurons, two layers of 32 hidden neurons each and a variable number of output neurons corresponding to the number of classes [7, 25]. However, the number of hidden layers as well as hidden units (HU) can be adapted. For comparison, this study employs one and two hidden layers with 32 hidden neurons each and the number of neurons as evaluated by cross validation for each model.

The Netlab MLP is also a feedforward ANN, but with scaled conjugate gradient as training algorithm. It consists of 32 input neurons, one hidden layer with 32 hidden neurons and variable output neurons. Because the Netlab architecture is based on Bishop [28], only one hidden layer is available. Output function used by the MLP was either softmax or logistic with a 0 to 1 output range.

Both training algorithms are limited to a maximum number of 200 iterations.

### D. Analysis

The Wilcoxon Sign-Rank test with a significance level of $\alpha$ = 0.05 is applied to all algorithms and their respective parameters to evaluate significance for each movement classification accuracy. The mean accuracy (Acc), standard deviation (SD) and p value of the models, as well as training time in seconds, will be given in the tables. The SD was calculated over the averaged iterations and across subjects and movements. Although training time is not regarded as an important aspect for offline classification, short training time has advantages in practical applications such as immediately available prosthesis control without a delay caused by the computing training algorithm. Fast training would also be a requirement for prospective online adaptive learning.

## Results

### A. Performance comparison of linear models

The direct comparison of accuracies for BioPatRec LDA and Netlab GLM can be found in Table 1. Although LDA and GLM are both linear models, GLM performs better because it is based on the training algorithms scg or irls. For a single output model, GLM scg achieved significantly higher accuracies than GLM irls, although the difference is small. As for models with multiple output, the GLM irls and GLM scg training algorithms performed equally well. Highest significant accuracies are marked italics in Table 1.

**Table 1:** Comparison of linear models

Outp. type	BioPatRec LDA		Netlab GLM netopt (scg)		Netlab GLM train (irls)	
	Acc	SD	Acc	SD	Acc	SD
Single	0.938	0.072	*0.974*	0.034	*0.971*	0.037
Multiple	0.789	0.178	*0.837*	0.140	*0.837*	0.139

*Italics* significantly higher accuracy against LDA with p < 0.01

GLM netopt single output has significantly higher accuracy than GLM train with single but not multiple outputs

### B. Optimal number of hidden units

**Fig. 1:**
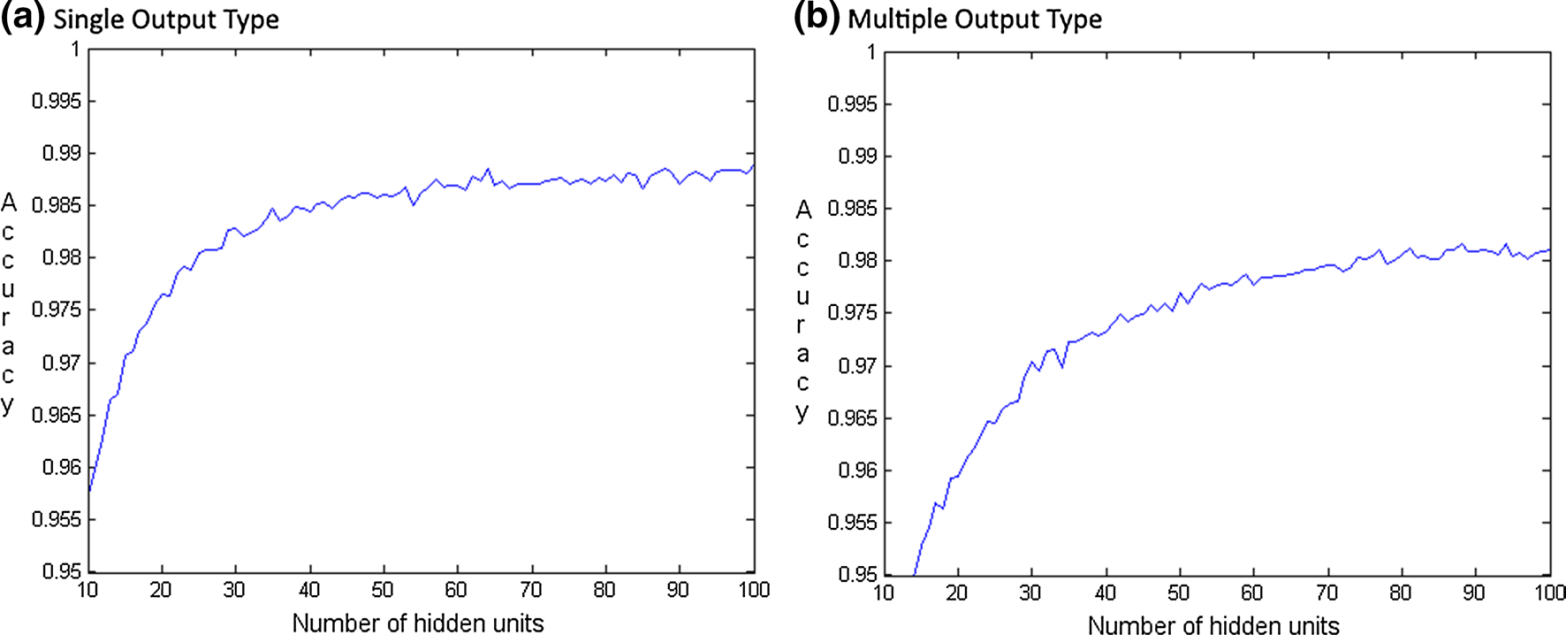
**a** Single output type: this* figure* shows the accuracy reached for every number of hidden units. After 64 HU there was no significant increase in accuracy. **b** Multiple output type: this* figure* shows the accuracy reached for every number of hidden units. After 74 HU there was no significant increase in accuracy

Considering that there was no indication in BioPatRec as to why two layers of 32 hidden units were used [20], an evaluation to find the optimal number of hidden units for each classification problem was performed. For each number of hidden units (1–100), 10 MLPs were trained and evaluated (cross-validation). The accuracy for each number of hidden units was averaged over those 10 MLPs and can be seen for simgle output type in Fig. 1a and multiple output type in Fig. 1b.

Because of time concerns, hidden unit evaluation was performed for Netlab. The Wilcoxon Sign-Rank test evaluated if an increase in the number of hidden units significantly improved the accuracy. The smallest number of hidden units out of 100 maximum permissible, for which no significantly higher accuracy could be observed are: (a) 64 hidden units for a single output type and (b) 74 hidden units for the multiple output type. However, from 30 to 40 hidden units on, the accuracy only slightly increases. The peaks seen on the curves cannot be attributed to statistical error but are due to systematic variance between the number of hidden units.

As the network for the multiple output (74 HU) problem is more complex than for single output (64 HU) it could be assumed that the multiple output problem is more complex.

### C. Performance comparison of neural networks

MLPs with different network complexities were tested within Netlab and BioPatRec and results are shown in Table 2. Since the highest classification accuracy per output type has tested significant against all other methods in BioPatRec and Netlab, it is marked italics within the table. The Netlab neural nets achieved significantly higher accuracies in single as well as in multiple output type. This is expected due to the better performance of the scg training algorithm compared to gradient descent. With an optimized number of hidden units, the accuracy of the Netlab MLPs could be additionally increased from 98.3 to 98.7 % in the single output type and from 97.0 to 98.0 % in the multiple output type. For the BioPatRec MLPs there is no higher accuracy for the optimized number of hidden units. This is probably due to the weak performance of gradient descent in higher complexity networks. Another finding is the better performance of single output type against multiple output type for the respective settings with highest accuracy, which was Netlab MLP with an optimized number of hidden units.

**Table 2:** Performance comparison of non-linear models and optimal number of hidden units (HU)

BioPatRec MLP	32 HU		2 × 32 HU		64 /74 HU	
Outp. type	Acc	SD	Acc	SD	Acc	SD
Single	0.941	0.062	0.951	0.054	0.923^a^	0.055
Multiple	0.954	0.047	0.949	0.049	0.953^b^	0.043

Netlab MLP	32 HU		64 /74 HU	
Outp. type	Acc	SD	Acc	SD
Single	0.983	0.024	*0.987* ^a^	0.019
Multiple	0.970	0.033	*0.980* ^b^	0.025

*Italics* highest accuracy is significant against all other accuracies for single/multiple output type with p < 0.01

^a^ 64 HU

^b^ 74 HU

### E. Training time 

Training time for linear models as well as artificial neural networks can be found in Table 3. Linear models train fastest and MLPs show a huge discrepancy between BioPatRec and Netlab, with Netlab MLP being 10^2^ times faster than BioPatRec MLP.

**Table 3:** Training time (in s)

	Model	Single output	Multiple output
BioPatRec	LDA	0.35	0.41
Netlab	GLM scg	1.07	1.23
	GLM irls	0.51	0.43
BioPatRec (gradient decent)	MLP 32HU	172	127
	MLP 2 × 32 HU	279	238
	MLP 64 HU	178	–
	MLP 74 HU	–	123
Netlab (scg)	MLP 32HU	1.88	1.38
	MLP 64 HU	2.49	–
	MLP 74 HU	–	3.08

## Discussion

Netlab’s GLM with irls training algorithm performed significantly better than BioPatRec’s LDA and still maintained short training time. The performance accuracies of BioPatRec’s MLP are best with two hidden layers of 32 neurons. However, Netlab’s MLP consisting of one layer of 32 hidden neurons performed significantly better. During testing of different training algorithms for MLP which all had a maximum number of iterations of 200, the results showed that the scg learning algorithm for MLP was superior in accuracy. These findings correspond to the results of previous benchmarkings of scg against the standard backpropagation, the conjugate gradient backpropagation and the quasi-Newton algorithm [30]. The Netlab MLP with optimized number of hidden units achieved 98.7 % accuracy compared to 95.1 % of the standard BioPatRec MLP, which in terms of error rate is an improvement from 4.9 to 1.3 %. When comparing normalizations for preprocessing standard normal distribution was found to be superior to a transformation to mid-range of 0 and range of 2.

Multiple output types do not work well with linear methods because they seem to require non-linear separation (as can be seen in the classification accuracies) and single output types perform well with linear methods, however, non-linear algorithms are still considerably superior. Single output type always performs better than multiple output type (with BioPatRec MLP being the only exception). This suggests that the single output type is a more simple solution to the same problem, although multiple output type has the advantage to recognize combined movements with only having the single movements available. Future research could focus on the issue of recognizing combined movements after training only single movements.

Regarding training time, Netlab was possibly faster because it is a sophisticated toolbox especially optimized for neural computation and its utilization of hyperthreading.

However, it is important to not only look at offline performances, but also to take into consideration that several factors challenge the robustness and reliability of pattern recognition algorithms in real-time. Electrode condition and displacement as well as sweat and muscle fatigue influence conductivity of the electrodes [31–34]. Therefore this study’s results will be incorporated into further movement classification assessments in real-time.

## Conclusions

Integrating Netlab’s efficient training algorithms for artificial neural networks and linear models into the BioPatRec environment resulted in an improvement of offline classification accuracies and training time. The Netlab toolbox for neural computation has been successfully implemented into the newest releaese of BioPatRec (v4.0).

## Abbreviations

ANN: artificial neural network
LDA: linear discriminant analysis
MLP: multi layer perceptron
GMM: Gaussian mixture model
GLM: general linear model
EMG: electromyography
HU: hidden units
Acc: accuracy
SD: standard deviation
scg: scaled conjugate gradient
irls: iteratively reweighted least squares
